# Obstructive Sleep Apnea in Developmental Age: 22-Item Pediatric Sleep Questionnaire for an Observational Descriptive Investigation

**DOI:** 10.3390/children10071265

**Published:** 2023-07-22

**Authors:** Francesca Cremonini, Ludovica Zucchini, Federica Pellitteri, Mario Palone, Luca Lombardo

**Affiliations:** Postgraduate School of Orthodontics, University of Ferrara, 44121 Ferrara, Italy; francesca.cremonini@edu.unife.it (F.C.); ludovica.zucchini@edu.unife.it (L.Z.); mario.palone@unife.it (M.P.)

**Keywords:** Sleep Apnea Syndrome, surveys and questionnaires, epidemiological monitoring, OSAS, pediatric dentistry

## Abstract

The aim of this paper is to perform an observational descriptive study of the Obstructive Sleep Apnea Syndrome (OSAS) in a population of children by evaluating the prevalence and role of sex and age variables. The 22-item Pediatric Sleep Questionnaire (PSQ) was administered to parents of children aged 3 to 12 years. The questionnaire is a very simple tool since it allows for the indication of patients with possible OSAS diagnosis through a cut-off of 0.33. The anonymous diagnostic questionnaire, available in digital format, was administered to the population under study by a link or QR code. Only the questionnaires completed in all their parts were recorded and analyzed. Eight hundred and thirty-two questionnaires were collected. One hundred and fifty-four subjects obtained a PSQ score > 0.33 and the prevalence of OSAS was 18.51%. The Chi-square test showed a statistically significant association between the PSQ score > 0.33 and male sex. The higher prevalence of subjects with a value of PSQ > 0.33 (n = 277) are aged 3–4–5 years, followed by the 6–7–8 range in the group with PSQ score > 0.33, *p* < 0.05. The prevalence of OSAS was 18.51% in children aged 3 to 12 years. The variables male biological sex and the age group 3–8 year were statistically significant for subjects with OSAS diagnosis. This study underlines the use of the 22-item Pediatric Sleep Questionnaire as a first screening tool to identify children at risk of OSAS.

## 1. Introduction

Obstructive Sleep Apnea Syndrome (OSAS) is a frequent pathology in the pediatric age and one of the most serious forms of sleep respiratory alteration in young patients.

OSAS is defined by the American Thoracic Society as a respiratory disorder of the upper airways, characterized by nocturnal episodes of complete intermittent and/or prolonged partial obstruction, which disrupt ventilation and physiological sleep patterns [1].

Most affected children have normal breathing during the day; a minority with severe upper airway obstruction can also have labored breathing when awake and noisy. OSAS children could manifest the following clinical signs: Snoring, restlessness during sleep, and chronic mouth breathing [1].

An easy indication for evaluation could be the loud snoring, which disturbs parents, but making an evaluation only using this parameter can be a mistake. Indeed, some infants with clinical OSAS have little or no snoring.

Daytime hypersomnolence, failure to thrive, cor pulmonale, frequency of behavior, enuresis, personality and learning problems may be observed [1].

Delayed diagnosis of this syndrome can lead to severe neurological and cardiopulmonary comorbidities and a worsening of the child’s quality of life [2].

Complications that an untreated pediatric severe OSAS may encounter are: Pulmonary hypertension, cor pulmonale, and right heart failure [2]. Today, early diagnosis has reduced these serious complications. However, it remains unclear whether these types of complications develop exclusively in severe cases of the syndrome, or current techniques do not allow for highlighting the problems in incipient or mild cases. Nevertheless, a vascular damage has already occurred.

Moreover, the prevalence in pediatric age is very heterogeneous and can range from 0.7% up to 24%, following the use of instrumental investigations, diagnostic criteria, and different populations analyzed [3,4]. Anatomical factors may have possible correlations to the increased risk of OSAS in young and adult patients [5,6,7].

Major OSAS children risk factors include hypertrophy of the neuromuscular disease including hypotonia and hypertonia conditions, tonsils and adenoids, obesity and genetic syndromes, such as Pierre Robin sequence and Down syndrome [1].

Sleeping disorders among children can be concomitant with other orofacial behaviors. In the current literature, factors which may lead to sleep fragmentation and night awakening have been significantly associated with sleep bruxism and TMD [8,9].

Clinical experience suggests that diagnosis, pathophysiology, clinical manifestations and management in suspected obstructive sleep apnea cases are different between children and adults [1].

Pediatric sleep questionnaires are non-invasive, effective, inexpensive tools when Polysomnography (PSG) is not available. Polysomnography, despite being the gold standard exam for diagnosing OSAS patients, is limited in its application by technical difficulties, long waiting times, and costs [10,11]. For these reasons, the questionnaires show themselves to be a valid alternative as the first diagnostic tool in order to identify children at risk of OSAS [12].

Currently, several questionnaires, such as the Obstructive Sleep Apnea-18 (OSA-18), the Children’s Sleep Habits Questionnaire (CSHQ), the Sleep Disturbance Scale for Children, and the 22-item Pediatric Sleep Questionnaire (PSQ), are available, most of which have been translated from English into other languages. Many of these questionnaires have been validated and translated from their original language into Italian, as well [13,14]. Among these, Italian guidelines [12] recommend the use of the 22-item questionnaire as the first screening tool in pediatric patients, based on the latest literature that highlights its high diagnostic accuracy [15,16].

A recent study on this subject, identifying the PSQ as a useful screening tool, points out that the OSAS prevalence is 10% in children between 6 and 12 years. The results underline that no statistically significant differences were found for the sex and age variables [17].

Given the heterogeneous prevalence values of the disease [4], its tendency to increase between 2 and 8 years and to decrease after 9 years of age [18,19], as well as the lack of clarity about the role of sex in OSAS; therefore, it is necessary to study the frequency of the disease, the influence of sex, and the age group of the patient. Furthermore, the current literature presents a small number of studies conducted through the PSQ questionnaire submission.

The questionnaire proposed should be a first screening step in order to address the OSAS patient in the correct diagnostic-therapeutic pathways.

This new investigation has the purpose of showing little and heterogeneous information on OSAS pediatric patients, which is currently present in the literature. This observational study, on an Italian children population, highlights the clinician’s crucial role in the diagnosis of this pathology, which is often forgotten and misunderstood.

Furthermore, this research shows how a questionnaire could be a fast and useful first screening tool and the importance of using it during the clinical practice using a paper or digital format.

In conclusion, the aim of this study was to perform an epidemiological survey to identify OSAS in a pediatric patient population by analyzing the prevalence and role of sex and age variables in the disease, through the administration of the Pediatric Sleep 22-item Questionnaire.

## 2. Materials and Methods

### 2.1. Study Design

An observational descriptive study was performed to analyze the OSAS in a pediatric population, through the administration of the Pediatric Sleep 22-item Questionnaire.

All subjects gave their informed consent for inclusion before they participated in the study. This observational study has been reported in accordance with the STROBE Statement checklist.

Structural validity of the study protocol was assessed using a previously validated questionnaire: 22-item PSQ. Chervin et al. [11] have demonstrated the validity and the reliability of PSQ scales and how PSQ could be a valid instrument in clinical research when polysomnography is not available [11].

### 2.2. Setting

The survey was conducted in a Caucasian population of children, recruited on a voluntary basis from September 2021 to April 2022 in Italy.

Children patients have been recruited thanks to some pediatricians, elementary schools, and dentists who made themselves available for proposing the questionnaire to the children’s parents. No previous clinical examination has been carried out in order to use the questionnaire as a first step of screening.

The anonymous diagnostic questionnaire, available in digital format, was administered to the population under study by a link or QR code. The parents/guardians of the children were asked to answer all the questions in the questionnaire.

It was not possible to organize a face-to-face sampling survey due to the pandemic situation. For this reason, an online survey form was designed using the program Google Forms (https://docs.google.com/forms/, accessed on 10 June 2021).

This online format of investigation was able to follow travel restrictions, distancing, and prevention guidelines to control the spread of COVID-19. This is in accordance with the rising number of studies based on online surveys since the beginning of COVID-19 pandemic [20].

The program used for the epidemiological investigation was Google Forms (https://docs.google.com/forms/, accessed on 10 June 2021), the program records only questionnaires completed in all their parts and automatically rejects the other ones. The structure of the program used prevents us from calculating the number of subjects which were excluded from the sample.

All participants were informed about data collection before starting to complete the questionnaire. No personal information will be collected except for: Biological sex and age of the subject. The information will be kept for the entire duration of the data collection on Google Forms platform; the data will not be used for commercial purposes but will be treated with respect to privacy. The Google Forms survey was supported by the University of Ferrara.

### 2.3. Participants

The inclusion criteria adopted were:

- The age range of the subjects analyzed: 3–12 years old.
- The presence of a smartphone or a device with an internet connection in the family.

On the other hand, the exclusion criteria adopted were:

- One or more missing answers among the 22 survey questions: Only patients who returned a questionnaire completed in all its parts were recorded and analyzed in the study. Patients with partial or incomplete questionnaire have been excluded from the investigation by Google forms platform setting.
- Italian children population: The questionnaire proposed has been written using Italian language; therefore, only Italian children have been included in the study.

### 2.4. Study Size

The survey was conducted in a Caucasian population of children, aged 3–12 years. The sample recruited consists of 832 children, 427 males, and 405 females.

### 2.5. Variables

All the variables assessed in the population, during the epidemiological screening investigation, are:

age range: 3–4–5 vs. 6–7–8 vs. 9–10–11–12;
biological sex: male vs. female;
PSQ score: >0.33 vs. <0.33.

### 2.6. Data Sources

The questionnaire is made up of the 22 questions belonging to the Pediatric Sleep Questionnaire [16], which allowed for the calculation of a PSQ score for each subject. Two other questions regarding biological sex and age variables were also integrated, which were not counted in each subject’s PSQ score, but were analyzed separately.

The 22-item PSQ formula was previously validated in Italian with a traditional paper format in Ranieri et al. [16] study.

In order to obtain the score of the questionnaire, it is necessary to perform a simple calculation according to the formula:

$$\mathrm{PSQ SCORE}=\frac{\mathrm{Number of affirmative answers}\;“\mathrm{Yes}”}{\mathrm{Number of affirmative answers}\;“\mathrm{Yes}”\;+\mathrm{Number of negative answer}\;“\mathrm{No}”}$$

The result obtained is a variable score from a minimum of 0.0 to a maximum of 1.0. According to what is defined in the article by Ranieri et al. [16], the cut-off adopted was a score of 0.33. Patients with PSQ scores > 0.33 were considered positive at OSAS diagnosis, while patients with scores < 0.33 were considered negative at diagnosis.

The accuracy level of this cut-off point is highlighted by Chervin et al. [11], this study suggests that the optimal scale cut-off to indicate the presence of OSAS would be 0.33 and greater values suggest the diagnosis. This criterion correctly classified 85–86.4% of the subjects and resulted in a test sensitivity range between 0.81 and 0.85 and a specificity of 0.87 [11].

### 2.7. Statistical Methods

The dataset consists of 832 children, in order to evaluate the margin of error and the confidence level due to the entire sample size and the number of patients with PSQ score > 0.33, the a posteriori formula is used. The a posteriori formula is used: $n=\frac{z^{2}\;\times \;p\;\left(1-p\right)}{e^{2}}$ where *z* is the *z*-score, *p* is the standard deviation, and e is the margin of error.

In regard to the sample size, following the application of the previous formula, for the total sample a 99% confidence interval and a margin of error of 0.03 has been used.

The univariate descriptive analysis of the study parameters was performed: Biological sex (male and female), age range (3–4–5, 6–7–8, 9–10–11–12), and PSQ score (>0.33, <0.33). The analysis reported the sample size and percentage value of the variables.

The homogeneity of the sample by sex and age was verified using the Chi-square test for a single sample.

In order to verify the association between the diagnosis of OSAS, positive or negative, in the patients under study and the sex and age parameters, a dichotomous variable was created to indicate whether the subject had obtained a PSQ score >0.33 or <0.33.

Any associations between the sex variables and the age range were verified between the groups with PSQ scores >0.33 or <0.33 and subsequently within each PSQ group, through Chi-square tests.

In all the analyses mentioned, an alpha significance level of 0.05 was used. For the statistical analysis of data, the IBM SPSS Statistics software was employed, which was carried out using frequency tables for the qualitative variables, and were then calculated in version 28.

## 3. Results

### 3.1. Participants

Following the registration of the surveys administered and completed in all their parts, a sample of 832 questionnaires was obtained, whose answers are described in Table 1.

The sample size calculation, based on a total population of 832 subjects, guarantees a confidence level of 99% and a margin of error of 0.03.

### 3.2. Descriptive Data

The descriptive analysis of the total population under study is represented by Table 2.

The population is homogeneous with respect to the sex variable (*p* = 0.446), while it is not homogeneous neither for the age variable (*p* < 0.001) (Table 3) nor for the dichotomous variable PSQ (>0.33, <0.33).

If, on the other hand, the total sample is analyzed according to the dichotomous variable PSQ < 0.33, >0.33 (Table 4), it can be observed how the sex and age variables are not homogeneous within the identified groups.

A prevalence of males in the group with a PSQ value > 0.33 compared to females and a higher prevalence of females in the group with a PSQ value < 0.33 is also underlined (Table 4).

### 3.3. Main Results

The Chi-square test shows a statistically significant association between the PSQ score > or <0.33 and biological sex (Table 5).

Moreover, a higher prevalence of subjects aged 9–10–11–12 years is reported in the group of patients with PSQ value < 0.33 (*n* = 277), while in the group of children with PSQ value > 0.33 the number is greater in the range 3–4–5 years (*n* = 58), followed by the range 6–7–8 years (Table 4). The analysis again reports a statistical significance with *p* < 0.05, which confirms a further association between the PSQ value > 0.33 or <0.33 and the age variable (Table 6).

The strength of statistically significant associations between PSQ value and sex/age was investigated thanks to the Cramer test. The value of Cramer’s V shows how the strength of the association is slight for both (Table 7).

## 4. Discussion

Although the gold standard examination for the diagnosis of OSAS is Polysomnography (PSG) [21], this examination has major limitations in growing patients. It is an often-selective procedure, and since it is not always available in the hospital, it involves a high cost and technical difficulties in carrying out the investigation in a pediatric patient. For these reasons, it has been shown that an anamnestic questionnaire can prove to be an effective screening method in the diagnosis of the pathology [22,23]. In particular, the questionnaire adopted in the present study is the Pediatric Sleep Questionnaire, in the form reduced to 22 items, according to the 2016 National Guidelines [8]. Furthermore, two recent meta-analyses have shown that the PSQ presents the highest sensitivity (74%), among the questionnaires examined for the evaluation of OSAS [10,15].

The analysis was performed by adopting a digital questionnaire. In accordance with COVID-19 sanitary recommendations, this online format of investigation was able to follow travel restrictions as well as distancing and prevention guidelines. Some studies have compared the results achieved with different survey administration modes (online survey vs. traditional paper format), with contrasting conclusions, suggesting large, small, or non-significant differences between the two different formats [24,25,26].

The prevalence of OSAS found in the study population was 18.51%. In particular, the subjects with scores > 0.33 were found to be 63.63% male and 37.66% aged between 3 and 5 years, highlighting a statistically significant heterogeneous sample for the variables with *p* < 0.05.

Sleep Relating Disorder (SDB) prevalence in pediatric age is various, as described by Lumeng et al. [27]. This analysis has reviewed the available evidences on the prevalence of OSAS among children, including 48 original research studies, and dissimilar results were found.

The significant heterogeneity of findings reported, by each study included in the review, could be justified by several demographic and anthropometric factors of the population considered. A lack of standardization of the OSAS diagnostic criteria adopted in clinical researches and of the population taken in exam, which differs by ethnicity, sex, age, and weight status, could explain the variety of prevalence obtained.

Moreover, a recent survey shows a high rate of 22.2% of possible obstructive sleep apnea syndrome in school-aged children in Damascus Syria [28]. The study has been conducted using a sleep questionnaire on a similar size population but in a different country, as compared to the current investigation.

On the other hand, Brunetti et al. [29] have performed an investigation on an Italian children population, reporting a OSAS prevalence of 1%, the lower values obtained are explained by the different study designs. The authors applied two diagnostic steps during the investigation, first a sleep questionnaire and second a polysomnographic evaluation. In the current survey, only a first screening test has been performed.

Few studies in the literature have realized a screening test on a population of the same ethnicity, size, and using the same diagnostic questionnaire (PSQ) and for these reasons, it is comparable with the epidemiological survey performed.

A survey of Paduano et al. [17], which met the majority of these criteria, reports a prevalence of OSAS in the pediatric age, which is equal to only 10% [17].

The difference in prevalence between the present study and that of Paduano et al. [17] is probably due to the different age ranges of the sample examined, as only children aged between 6 and 12 years are included. In fact, the lack of inclusion of subjects between 3 and 5 years of age could have influenced the lower prevalence value, as it seems to be the age group most affected by the disease [18,21].

In the group of subjects who obtained a PSQ score > 0.33, and thus positive at the diagnosis of OSAS, the results report a statistically significant prevalence in males. A meta-analysis conducted by Lumeng et al. [27] points out that some studies have shown a higher prevalence of respiratory disorder symptoms in male subjects, while other studies have reported no differences between the sexes. Surveys that reported higher prevalence in males analyzed large populations of subjects [30], compared to studies in which biological sex differences were not relevant (*n* = 190) [31]. Furthermore, in accordance with the results reported in the following study, a survey that evaluates the prevalence of OSAS in a population of 6447 patients proves relevant; male sex was statistically associated with OSAS pathology [32]. This may suggest that the differences between the sexes are not always detected, probably due to an insufficient sample size, which therefore does not allow for the observation of a statistically significant difference.

The results of the following study report a statistically significant association between PSQ score > 0.33 or <0.33 and age. In particular, it is highlighted that in the age group 9–10–11–12 years, the subjects with PSQ scores < 0.33 are more numerous, while the subjects diagnosed with OSAS pathology are more numerous in the 3–4–5 year and the 6–7–8 year age groups. The results obtained, in accordance with the conclusions of the study by Tan et al. [18], show how a peak prevalence of OSAS occurs in the age group 3–8 years. These results seem to be supported by anatomical reasons: In the age group 2–8 years, the upper airways are narrower, while the adenoids and tonsils increase in volume and the soft palate overlaps the epiglottis, predisposing the child to obstructive episodes, which decrease with growth thanks to the sagittal and vertical growth of the mandible and to a continuous increase in the volume of the upper airways [33].

Despite the statistically significant results obtained, the study presents a limit represented by the reduced sample size in reference to the Italian population investigated. Further studies with a larger sample are needed in order to verify the real prevalence of OSAS disease within the pediatric population between 3 and 12 years of age.

Moreover, general health information of study participants has not been examined before the administration of the Pediatric Sleep Questionnaire. The focus of this investigation is to evaluate the prevalence of OSAS in a wide population and to perform a first fast screening of the pathology, which can be easily applied in the clinical practice. Future studies could analyze the possible correlation between previous pathologies and OSAS in a pediatric population.

It would also be desirable to investigate the role of the dentist and pediatrician within the early diagnosis of the disease.

On the other hand, the lack of standardization of clinical studies, aimed at evaluating the prevalence of OSAS in developmental age, is evident in the literature. The lack of universally accepted diagnostic criteria and tests and the lack of homogeneity in the populations under study make it difficult to obtain a correct assessment of the prevalence of the disease. For these reasons, the use of the 22-item Pediatric Sleep Questionnaire, as the first step in the diagnostic setting of the pediatric patient, could be an inexpensive, quick, and non-invasive solution for diagnosing young patients.

This questionnaire should be a daily used tool in clinical practice, helping and facilitating in OSAS diagnosis. Despite the high prevalence of this syndrome reported in this survey, this pathology is often forgotten by many clinicians. The PSQ could be a solution to execute a first screening, wasting little time and resources. It can be administered during the appointments or also at home, in paper or digital format, addressing the OSAS patient into the correct diagnostic-therapeutic pathways.

Other future researches on this topic are certainly necessary, in order to shed light on this pathology in pediatric patients whose etiopathology, implications, and treatment are not yet known and standardized.

## 5. Conclusions

Based on the results that emerged during the epidemiological investigation conducted through the 22-item Pediatric Sleep Questionnaire, we can conclude that:

1. The prevalence of OSAS in pediatric age was 18.51%.
2. The presence of a statistically significant association between the PSQ value > 0.33 and the male sex compared to the female was highlighted.
3. An association between PSQ value and age is reported, in particular, subjects with PSQ scores < 0.33 in the 9–10–11–12 age group and subjects with PSQ scores > 0.33 in the age group were significantly more numerous in the age groups of 3–4–5 and 6–7–8 years.

## Figures and Tables

**Table 1:** Percentage and number of answers obtained to the 22-item “Pediatric Sleep Questionnaire”.

		No	I Do Not Know	Yes	Total
While sleeping, does your child:					
Snore more than half of the time?	**%**	87.1	3.7	9.1	100
	*n*	725	31	76	832
Always snore?	**%**	95.2	1.9	2.9	100
	*n*	792	16	24	832
Snore loudly?	**%**	90.5	1.1	8.4	100
	*n*	753	9	70	832
Have “heavy” or loud breathing?	**%**	72.7	1.7	25.6	100
	*n*	605	14	213	832
Have trouble breathing or struggle to breathe?	**%**	90.3	3.2	6.5	100
	*n*	751	27	54	832
Have you ever seen your child stop breathing during the night?	**%**	66.7	13.3	20.0	100
	*n*	555	111	166	832
Does your child:					
Tend to breathe through the mouth during the day?	**%**	76.0	4.4	19.6	100
	*n*	632	37	163	832
Have a dry mouth on waking up in the morning?	**%**	63.2	12.1	24.6	100
	*n*	526	101	205	832
Occasionally wet the bed?	**%**	80.4	1.0	18.6	100
	*n*	669	8	155	832
Wake up feeling unrefreshed in the morning?	**%**	75.8	5.2	19.0	100
	*n*	631	43	158	832
Have a problem with sleepiness during the day?	**%**	90.3	1.2	8.5	100
	*n*	751	10	71	832
Has a teacher or other supervisor commented that your child appears sleepy during the day?	**%**	94.4	1.8	3.8	100
	*n*	785	15	32	832
Is it hard to wake your child up in the morning?	**%**	59.7	0.6	39.7	100
	*n*	497	5	330	832
Did your child stop growing at a normal rate at any time since birth?	**%**	92.1	5.0	2.9	100
	*n*	766	42	24	832
Did your child stop growing at a normal rate at any time since birth?	**%**	91.8	2.8	5.4	100
	*n*	764	23	45	832
Is your child overweight?	**%**	89.8	1.7	8.5	100
	*n*	747	14	71	832
This child often:					
Does not seem to listen when spoken to directly?	**%**	73.9	1.1	25.0	100
	*n*	615	9	208	832
Has difficulty organizing tasks and activities?	**%**	75.2	5.2	19.6	100
	*n*	626	43	163	832
Is easily distracted by extraneous stimuli?	**%**	47.1	1.9	51.0	100
	*n*	392	16	424	832
Fidgets with hands or feet or squirms in seat?	**%**	71.4	1.1	27.5	100
	*n*	594	9	229	832
Is “on the go” or often acts as if “driven by motor”?	**%**	75.4	3.2	21.4	100
	*n*	627	27	178	832
Interrupts or intrudes on others (e.g., butts into conversations or games)?	**%**	75.4	3.2	21.4	100
	*n*	627	27	178	832

**Table 2:** Sample demographics of the total population.

	*n*	%
Population	832	100
Sex		
Male	427	51.32
Female	405	48.68
PSQ score		
<0.33	678	81.49
>0.33	154	18.51
Age		
3–4–5	268	32.21
6–7–8	244	29.33
9–10–11–12	320	38.46

**Table 3:** One-sample Chi-square test for variable age.

Total N	832
Test Statistic	35,909
Degree Of Freedom	9
Asymptotic Sig. (two-sided test)	<0.001

**Table 4:** Sample demographics of PSQ score.

	*n*	%
<0.33	678	100
Sex		
Male	329	48.53
Female	349	51.47
Age		
3–4–5	210	30.97
6–7–8	191	28.17
9–10–11–12	277	40.86
>0.33	154	100
Sex		
Male	98	63.63
Female	56	36.36
Age		
3–4–5	58	37.66
6–7–8	53	34.42
9–10–11–12	43	27.92

**Table 5:** Chi-square test for PSQ score and sex.

	Value	df	Asymptotic Significance (Two-Sided)	Exact Sig. (Two-Sided)	Exact Sig. (One-Sided)
Pearson Chi-Square	11.471	1	<0.001		
Continuity Correction	10.874	1	<0.001		
Likelihood Ratio	11.609	1	<0.001		
Fisher’s Exact Test				<0.001	<0.001
No. of Valid Cases	832				

**Table 6:** Chi-square test for PSQ score and age.

	Value	df	Asymptotic Significance (Two-Sided)
Pearson Chi-Square	8.870	2	0.012
Likelihood Ratio	9.184	2	0.010
No. of Valid Cases	832		

**Table 7:** Cramer test for sex and age.

SEX		Value	Approximate Significance
Nominal by Nominal	Phi	−0.117	<0.001
	Cramer’s V	0.117	<0.001
No. of Valid Cases		832	
**AGE**		**Value**	**Approximate Significance**
Nominal by Nominal	Phi	0.103	0.012
	Cramer’s V	0.103	0.012
No. of Valid Cases		832	

## Data Availability

All authors assured that all data and materials as well as software application or custom code support their published claims and comply with field standards. Data can be requested from the corresponding author at dott.lulombardo@gmail.com.
